# DNA Methylation and Transcript Variant Analysis of *CDKN2A* Exon 2 Despite High Sequence Identity with *CDKN2B* Exon 2

**DOI:** 10.3390/ijms26136128

**Published:** 2025-06-26

**Authors:** Katja Zappe, Andreas Jenik, Daniel Berger, Lukas Uhlik, Petra Heffeter, Margit Cichna-Markl

**Affiliations:** 1 Department of Analytical Chemistry, Faculty of Chemistry, University of Vienna, 1090 Vienna, Austria; 2 Center for Cancer Research and Comprehensive Cancer Center, Medical University of Vienna, 1090 Vienna, Austria

**Keywords:** *CDKN2A*, *CDKN2B*, p16^INK4a^, exon 2, DNA methylation, promoter, transcript variants, pyrosequencing, high-resolution melting, sequence identity

## Abstract

The tumor suppressor p16^INK4a^, encoded by *CDKN2A*, is frequently inactivated in cancer through genetic or epigenetic mechanisms. While promoter hypermethylation is the most common epigenetic cause, aberrant methylation of *CDKN2A* exon 2 has also been associated with various tumor types. However, analyzing DNA methylation of exon 2 is challenging due to its high sequence similarity with *CDKN2B*. We developed a pyrosequencing assay to analyze CpGs in *CDKN2A* exon 2, which was previously found to be hypermethylated in breast cancer. Our novel primer set enabled co-amplification of the homologous regions in *CDKN2A,* including CpGs 1–24, and *CDKN2B* CpGs 1–23. By quantifying the proportion of *CDKN2A*, we could accurately determine methylation levels for CpGs in *CDKN2A* exon 2. This method was applied to patient-derived glioma cells and commercial breast cancer cell lines. To reveal the role of exon 2 methylation in gene regulation, we additionally examined *CDKN2A^INK4a^* promoter methylation and expression at both mRNA and protein levels in breast cancer cell lines. We observed a range of (epi)genetic alterations, including homozygous deletions, transcript-specific expression, and exon 2 skipping. Our findings indicate that both promoter and exon 2 methylation contribute to regulation of *CDKN2A* expression. This novel method provides a valuable tool for future studies seeking a deeper understanding of *CDKN2A* regulation in cancer.

## 1. Introduction

Cyclin-dependent kinases (CDKs) play a crucial role in cell cycle regulation by tightly controlling progression from one cell cycle phase to another. The activity of CDKs is modulated by cyclins, which function as cofactors of CDKs, and by CDK inhibitors (CDKIs) [1]. The CDKI p16^INK4a^, for example, inhibits CDK4/6 (INK4, inhibitor of CDK4/6) by inducing a structural change in the cyclin binding site of CDK4/6, resulting in inhibition of the G1/S phase transition of the cell cycle. When p16^INK4a^ is inactivated, CDK4/6 binds to cyclin D, driving the G1/S phase transition of the cell cycle. Uncontrolled cell division due to cell cycle deregulation is a hallmark of human cancer [1]. Inactivation of p16^INK4a^ has been reported for a variety of cancer types, including glioblastoma [2], breast cancer [3], and non-small-cell lung cancer [4].

*CDKN2A* (*CDK inhibitor 2A*), the gene encoding p16^INK4a^, is located on chromosome 9q21. In addition to p16^INK4a^, the *CDKN2A* gene generates several transcript variants differing in their first exons, including p12^INK4a^ and p14^ARF^ (ARF, alternative reading frame) [5].

Inactivation of p16^INK4a^ can occur through genetic and epigenetic mechanisms. Genetic mechanisms include homozygous deletion of the p16^INK4a^ locus and loss-of-function mutations within coding regions [6]. Hypermethylation of the *CDKN2A^INK4a^* promoter, the most frequently described epigenetic aberration of *CDKN2A*, has been found to silence *CDKN2A* in various cancer types [7,8]. However, in breast cancer, the situation remains unclear. Some studies report a higher frequency of *CDKN2A^INK4a^* promoter methylation in breast tumors [9,10,11,12,13], whereas others have not found a difference between malignant and non-malignant breast tissues [14].

In addition to the *CDKN2A^INK4a^* promoter, *CDKN2A* exon 1α and exon 2 have been targets of DNA methylation analysis [15]. Hypermethylation of *CDKN2A* exon 2 has been linked to late-stage esophageal cancer, with *CDKN2A* exon 2 being methylated in eight out of 16 esophageal tumors but in none of 16 normal tissue samples. The methylation frequency was higher in late-stage (III and IV) than in early-stage tumors [15]. By targeting the *CDKN2A^INK4a^* promoter and *CDKN2A* exon 2 in breast cancer samples, Spitzwieser et al. found exon 2 to be more frequently methylated than the promoter [16]. Moreover, the methylation status of exon 2 was significantly higher in tumors than in tumor-adjacent and tumor-distant tissues from the same patients and in normal breast tissues from healthy women. In addition, *CDKN2A* exon 2 hypermethylation was associated with the molecular subtype of the tumor [16].

However, the high-resolution melting (HRM) assay for exon 2 used in the study by Spitzwieser et al. is not specific for *CDKN2A* exon 2. Due to high sequence identity between *CDKN2A* and *CDKN2B*, the HRM assay previously developed [16] yields the average methylation status of *CDKN2A* exon 2 and *CDKN2B* exon 2. *CDKN2B*, located within the same 35 kb multigene region as *CDKN2A*, encodes p15^INK4b^ and p10^INK4b^.

In this study, we aimed to develop a pyrosequencing (PSQ) assay to enable specific DNA methylation analysis of *CDKN2A* exon 2. The target region should include CpGs 5–14 of *CDKN2A* exon 2, previously analyzed by HRM [16]. Our strategy was to co-amplify the target region for *CDKN2A* and *CDKN2B* using a novel primer set. Within the target region, we attempted to identify positions that allow evaluation of the proportion of *CDKN2A* and thus accurately determine DNA methylation levels for *CDKN2A* exon 2. To demonstrate the applicability of our approach, patient-derived tumor cells from glioma patients (sample set 1) and commercial breast cancer cell lines (sample set 2) were analyzed. With the second sample set, we also aimed to elucidate how *CDKN2A* exon 2 methylation is associated with *CDKN2A* expression. Thus, we also performed DNA methylation analysis of the *CDKN2A^INK4a^* promoter by using a novel primer set designed in-house. In addition, we designed primer sets enabling transcript variant analysis of *CDKN2A* exon 2 despite the high sequence identity with *CDKN2B* exon 2.

## 2. Results and Discussion

**Figure 1:**
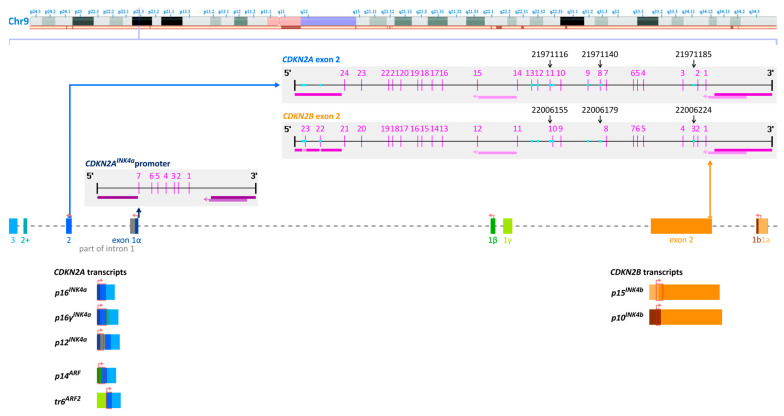
Chromosome 9 upper strand showing the *CDKN2A* (blue) and *CDKN2B* (orange) locus and the respective transcripts. For DNA methylation analysis, the seven CpGs directly upstream of exon 1α in the *CDKN2A^INK4a^* promoter and 24 CpGs of *CDKN2A* exon 2/21 CpGs of *CDKN2B* exon 2 were targeted. Pink vertical lines indicate CpG positions. PCR primers are indicated by upper horizontal bars (promoter: dark purple, exon 2: dark pink) and sequencing primers by lower bars (promoter: light purple, exon 2: light pink; arrow shows the sequencing direction). Differences between *CDKN2A* and *CDKN2B* exon 2 in the target region are highlighted by squares (cyan); three of them are also mispriming sites of *CDKN2B* for the reverse primer (highlighted). Black arrows show the three genomic positions allowing quantification of *CDKN2A* and *CDKN2B* PCR products by PSQ. Transcripts: horizontal bars show their exon composition, light red arrows translation starts, and light red box the coding sequence (CDS). The representation of chromosome 9, including the *CDKN2A*/*CDKN2B* transcript location, was taken from NCBI Genome Data Viewer [17], GRCh38.p14 primary assembly. The CpG line schemes were generated using Methyl Primer Express Software v1.0 (Thermo Fisher Scientific) and adapted manually.

Figure 1 shows a scheme of chromosome 9, including the positions of the *CDKN2A^INK4a^* promoter, the second exons of *CDKN2A* (blue) and *CDKN2B* (orange), the CpGs in these regions, and respective transcript variants. However, all transcripts of *CDKN2A* and *CDKN2B* result from the lower strand.

### 2.1. DNA Methylation Analysis of CDKN2A Exon 2—Method Development and Validation

#### 2.1.1. Primer Design and Characteristics of the Target Region

Designing primers for specific DNA methylation analysis of *CDKN2A* exon 2 was particularly challenging due to the high sequence identity between the second exons of *CDKN2A* and *CDKN2B* (254/307 bp, 83%, referring to non-bisulfite converted DNA). The target region should include CpGs 5–14 of *CDKN2A* exon 2, since these CpGs were previously found to be aberrantly methylated in breast cancer samples [16]. Due to the sequence similarity between the second exons of *CDKN2A* and *CDKN2B*, we anticipated that PCR might generate products for both genes. To cope with this problem, we tried to identify positions within the target region that would allow us to estimate the ratio of PCR products derived from *CDKN2A* and *CDKN2B* exon 2. By using the proportion of PCR products resulting from *CDKN2A* exon 2, one should be able to reliably determine the DNA methylation status of *CDKN2A* exon 2 despite the high sequence identity with *CDKN2B* exon 2.

**Figure 2:**
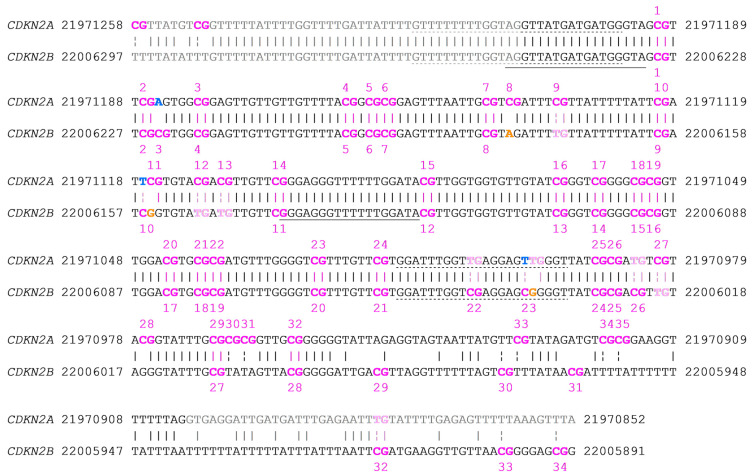
Sequence alignment of the bisulfite converted lower strand of chromosome 9 *CDKN2A* exon 2 (black), plus 50 bp up- and downstream (gray), respectively, with *CDKN2B*, using NCBI blastn. Alignment was annotated using coordinates from GRCh38.p14 primary assembly and highlighted manually. CpGs (pink; numbered according to their position in *CDKN2A* exon 2). PCR primers are indicated by dotted horizontal lines, sequencing primers by straight horizontal lines. C-to-T transitions affecting CpGs are indicated by dashed vertical lines. Bases specific for *CDKN2A* and *CDKN2B* are highlighted in blue and orange, respectively.

The region including CpGs 5–14 targeted by the novel primer set (Table 1) has a sequence identity of 95% (219/230 bp), referring to non-bisulfite converted DNA. The differences between the sequences are due to seven cytosine (C)-to-thymine (T) transitions, two C-to-adenine (A) transversions, one C-to-guanine (G) transversion, and one G-to-T transversion. However, since DNA methylation analysis by PSQ includes a bisulfite conversion step, the sequence identity after bisulfite conversion was more relevant. Following bisulfite conversion (of the lower DNA strand), the sequence identity between *CDKN2A* and *CDKN2B* exon 2 was 98% for originally methylated DNA and 96% for originally unmethylated DNA. In the bisulfite converted target region, *CDKN2A* and *CDKN2B* exon 2 differ at ten positions, all involving CpGs. Of these, six differences result from C-to-T transitions, while two involve C-to-A transversions. In the remaining two cases, base replacements led to either a shift in the CpG position or the formation of a new CpG (Figure 2).

The reverse primer (Table 1) showed several mismatches for *CDKN2B* exon 2, one (chr9:22006036) for originally unmethylated and three (chr9:22006036, chr9:22006037, and chr9:22006044) for originally methylated DNA (Figure 1 and Figure 2). Two mismatches occur at CpGs (in *CDKN2B*, Figure 1), where in *CDKN2A*, the CpG is replaced by TG.

The two C-to-A transversions (chr9:21971185 and chr9:22006179) are characteristic for *CDKN2A* and *CDKN2B* exon 2, respectively (Figure 1 and Figure 2). The third difference (chr9:22006155) in the sequenced region results from the shift of the position of the CpG.

**Figure 3:**
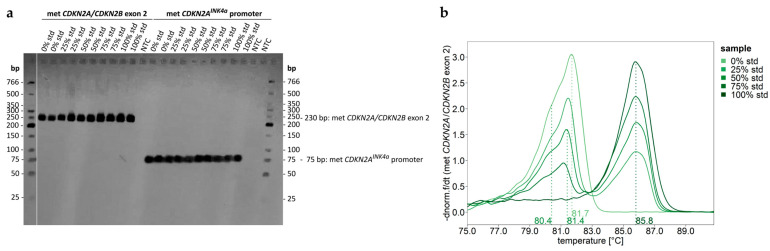
Verification of the formation of correct PCR products. (**a**) Agarose gel indicating that PCR products obtained for *CDKN2A*/*CDKN2B* exon 2 and *CDKN2A^INK4a^* promoter had the expected length. (**b**) Negative derivative of normalized HRM curves obtained for DNA standards (0%, 100% methylated) and mixtures thereof. Target region: *CDKN2A*/*CDKN2B* exon 2. Vertical dotted lines indicate the distinct melting temperatures. Together with data from PSQ, melting temperatures from left to right were assigned to unmethylated *CDKN2B* exon 2 (80.4 °C) and *CDKN2A* exon 2 (81.4 °C) alleles present in the 0–75% standards and fully methylated *CDKN2A* exon 2 (85.8 °C) alleles in the 25–100% standards. NTC: no template control; standards (std): non-methylated (0%) DNA, methylated (100%) DNA, and 25%, 50%, and 75% mixtures thereof.

PCR products for unmethylated (0%) DNA, methylated (100%) DNA, and mixtures thereof (25%, 50%, 75%) were subjected to agarose gel electrophoresis. PCR products obtained for *CDKN2A* and *CDKN2B* exon 2 were expected to be 230 bp long. Figure 3a indicates that PCR products of the correct length were obtained.

#### 2.1.2. Evaluation of Reliable DNA Methylation Levels Despite High Sequence Identity Between the Second Exons of CDKN2A and CDKN2B

Since HRM is highly suitable for detecting the co-occurrence of different alleles [18], PCR products were subjected to HRM before they were used for PSQ. HRM analysis of the PCR products obtained for 0%, 25%, 50%, 75%, and 100% methylated DNA standards resulted in the negative derivative of normalized HRM curves shown in Figure 3b. HRM curves obtained for DNA standards indicate that for the unmethylated standard, products for *CDKN2A* and *CDKN2B* exon 2 were obtained. In case of the 100% methylated DNA standard, the proportion of *CDKN2B* was too low and thus did not result in a shoulder.

**Figure 4:**
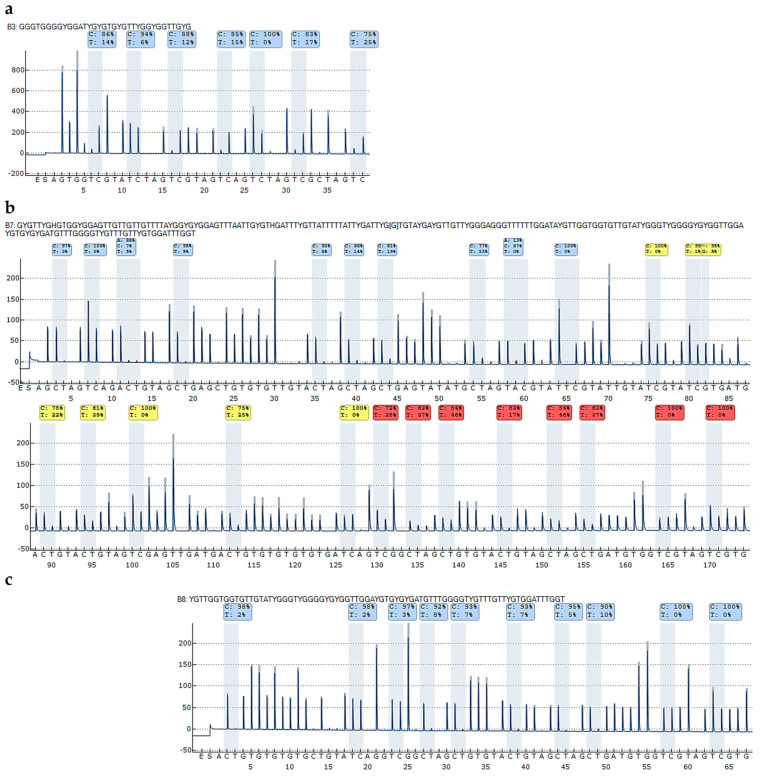
Representative pyrograms for the methylated (100%) DNA standard. (**a**) *CDKN2A^INK4a^* promoter CpGs 1–7 and (**b**,**c**) *CDKN2A*/*CDKN2B* exon 2 with (**b**) sequencing primer 1, targeting CpGs 1–24/CpGs 1–21, and (**c**) sequencing primer 2, targeting CpGs 15–24/CpGs 12–21. Peaks highlighted in blue–grey indicate the position to analyze; grey bars represent the expected heights according to the dispensation order (histogram). Nucleotide frequencies are shown above the respective position to analyze. Blue, yellow, and red colors indicate the quality of the result (blue: passed, yellow: check, red: failed).

Figure 4b,c show representative pyrograms, obtained for the 100% methylated DNA standard. By using only one sequencing primer (Figure 4b), DNA methylation levels of variable positions 19–26 could not be determined accurately since the signal to noise ratio was too low. This problem could be solved by applying a second sequencing primer (Figure 4c).

**Figure 5:**
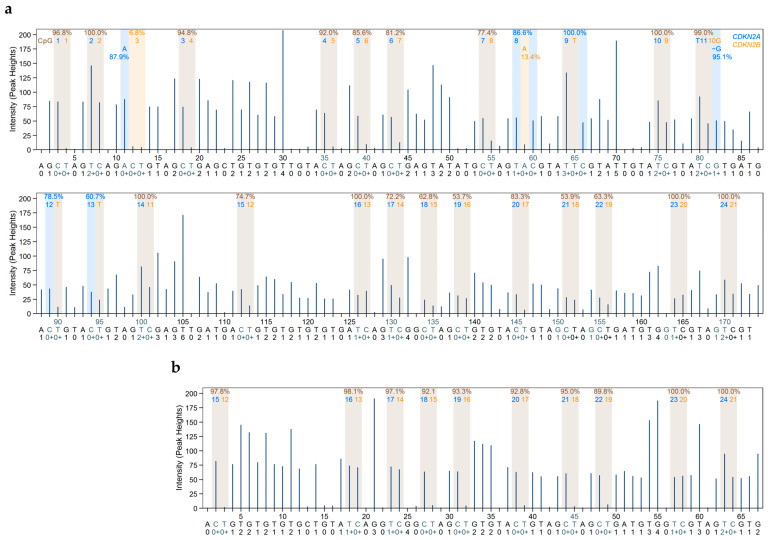
Translation of the position to analyze from pyrograms for *CDKN2A*/*CDKN2B* exon 2 (**a**) with sequencing primer 1 targeting CpGs 1–24/CpGs 1–21 and (**b**) with sequencing primer 2 targeting CpGs 15–24/CpGs 12–21 to the respective CpG (methylation shown above the region highlighted in brown or specific bases of *CDKN2A* (blue) and *CDKN2B* (orange)). Peak heights obtained from PSQ were plotted against the dispensation order and number of nucleotides incorporated. Data shown for the methylated (100%) standard.

Figure 5 shows the translation of the variable positions in the pyrogram to the respective CpGs in *CDKN2A* and *CDKN2B* exon 2. In addition, it indicates the positions (chr9:21971185, chr9:22006179, and chr9:22006155, Figure 2) that are specific for *CDKN2A* exon 2 or *CDKN2B* exon 2.

We tested these three variable positions for their suitability to indicate the ratio of PCR products derived from *CDKN2A* and *CDKN2B* exon 2. Position (pos) 3 A (*CDKN2A*; chr9:21971185) and pos 9 A (*CDKN2B;* chr9:22006179) show the respective ratio (Figure 1 and 2). At pos 13, there are two Gs in *CDKN2B* exon 2 (chr9:22006155–22006154) but only one G in *CDKN2A* exon 2 (chr9:21971115). This position (chr9:22006155/chr9:21971116) turned out to be less suitable, and thus, the proportion of *CDKN2A* was calculated from pos 3 and pos 9.

**Figure 6:**
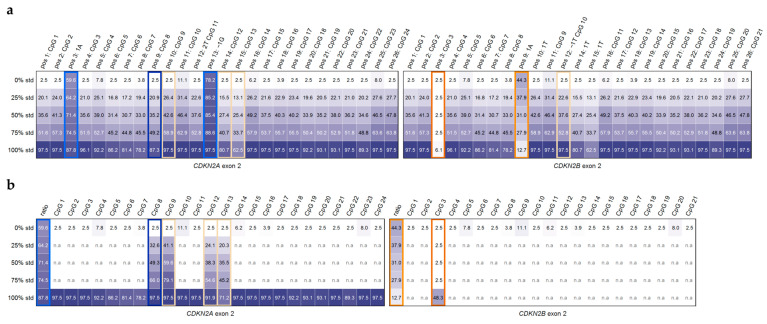
*CDKN2A*/*CDKN2B* exon 2 methylation levels of DNA standards sorted by their expected methylation status. DNA standards (std): non-methylated (0%) DNA, methylated (100%) DNA, and 25%, 50%, and 75% mixtures thereof. (**a**) Methylation status of the respective variable position (pos) in the pyrogram was assigned to the respective CpGs/alternative nucleotides. (**b**) Resulting heatmap for *CDKN2A*/*CDKN2B* exon 2 showing the specific methylation levels. n.a.: not analyzable due to simultaneous measurement of *CDKN2A* and *CDKN2B*. Specific positions for *CDKN2A* (blue frames) and *CDKN2B* (orange frames); for alternative nucleotides (lighter color) and CpGs (darker color). DNA methylation levels of specific CpGs were manually corrected by the proportion of CDKN2A/CDKN2B exon 2. Positions (ocher frame) were adjusted manually due to alternative T nucleotides.

The DNA methylation levels for 0%, 25%, 50%, 75%, and 100% methylated DNA standards, obtained by our approach, are given in Figure 6a. For pos 9 CpG 8 (chr9:21971140), which is specific for *CDKN2A*, our results indicate that in the presence of a higher proportion of *CDKN2B*, the results have to be corrected by eliminating the contribution of *CDKN2B* (Figure 6a,b). The correct DNA methylation level of the respective CpG in *CDKN2A* exon 2 is obtained by multiplying the DNA methylation given by 100 and dividing it by the proportion of *CDKN2A* exon 2 (pos 3 A, chr9:21971185). These corrections are necessary for pos 10 CpG 9, pos 14 CpG 12, and pos 15 CpG 13 because the DNA methylation status given refers to *CDKN2A*, whereas the DNA sequence of *CDKN2B* contains a T.

Similarly, the methylation level of pos 3 CpG 3 (chr9:22006224), which is specific for *CDKN2B*, has to be corrected by the proportion of *CDKN2B* exon 2 (pos 9 A, chr9:22006179).

The corrected methylation levels indicate that unmethylated strands are preferentially amplified in case both unmethylated and methylated strands are present for *CDKN2B*, as it is the case in DNA standard mixtures. Even in the methylated (100%) standard, a low number of unmethylated strands was amplified due to the three mismatches of the reverse primer with methylated strands.

However, the specific methylation status of the other CpGs could not be assigned to either *CDKN2A* or *CDKN2B* for the 25%, 50%, and 75% DNA methylation standard mixtures.

### 2.2. DNA Methylation Analysis of the CDKN2A^INK4a^ Promoter—Method Development and Validation

Since we were interested in elucidating the role of DNA methylation of *CDKN2A* exon 2 in gene expression, we also determined the DNA methylation status of the *CDKN2A^INK4a^* promoter. We designed a novel primer set (Table 1) that is specific for the *CDKN2A^INK4a^* promoter. Subjecting PCR products obtained with this primer set for unmethylated and methylated DNA standards and mixtures thereof to agarose gel electrophoresis indicated that PCR products of the correct length (75 bp) were obtained (Figure 3a). Figure 4a shows a representative pyrogram obtained for the 100% methylated DNA standard. Since the target region, containing seven CpGs, was rather short, it could be analyzed with one sequencing primer.

### 2.3. Transcript Variant Analysis of CDKN2A Exon 2—Method Development and Validation

**Figure 7:**
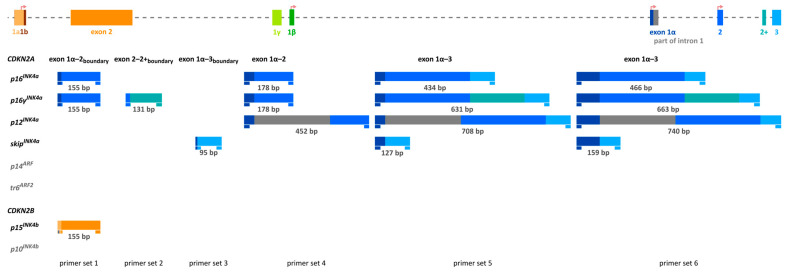
Lower strand showing the PCR products for the different transcript variants obtained with primer sets 1–6 (left to right). Bar colors symbolize the corresponding exons. The specific binding sites for exon–exon boundary-specific primer sets 1–3 (Table 2) and for primer sets 4–6 not targeting exons (Table 3) are shown below the transcript bars in the same color as the exon site. 5′-end of forward primer from primer set 1 targeting the *CDKN2A* exon 1α–exon 2 boundary is mispriming (dark blue) with *CDKN2B* exon 1a.

In order to investigate the association of DNA methylation of *CDKN2A* exon 2 with gene expression, we aimed at detecting *CDKN2A^INK4a^* transcripts that contain *CDKN2A* exon 2 as well as exon 2 skipping. We designed six primer sets, with primer sets 1–3 (Table 2) targeting *CDKN2A* exon–exon boundaries and primer sets 4–6 (Table 3) targeting *CDKN2A* exons. The high number of primer sets was necessary due to the variety of *CDKN2A^INK4a^* transcripts that can be generated (Figure 1). The PCR products for the different transcript variants obtained with primer sets 1–6 are shown in Figure 7.

### 2.4. DNA Methylation Analysis of CDKN2A^INK4a^ Promoter and CDKN2A Exon 2 in Primary Cell Lines from Glioma

We applied our primer set for *CDKN2A* exon 2 methylation to a sample set comprising 27 primary cell lines (PCLs) from glioma (sample set 1). To obtain a more comprehensive view of *CDKN2A* methylation, we also determined the methylation status of the *CDKN2A^INK4a^* promoter. Unfortunately, no RNA was available from this sample set, precluding further analysis of the functional relevance of *CDKN2A* exon 2 methylation.

**Figure 8:**
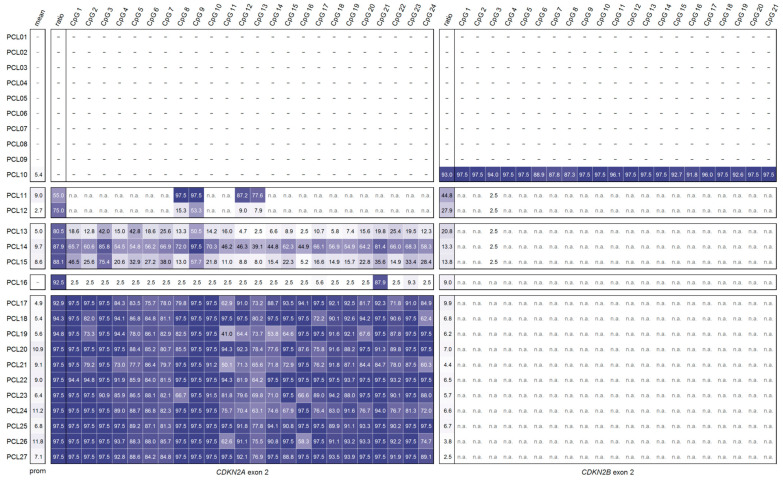
Heatmap for the *CDKN2A^INK4a^* promoter (HRM) and *CDKN2A*/*CDKN2B* exon 2 (PSQ) methylation levels of primary cell lines. —not present/detected; n.a.: not analyzable due to simultaneous determination of methylation levels of *CDKN2A* and *CDKN2B*.

In nine PCLs (PCL01–PCL09), no PCR products were obtained for either the promoter or exon 2 (Figure 8). We hypothesize that the absence of PCR amplification for both regions is due to deletion. Homozygous deletion of the *CDKN2A* gene, or even larger parts of chromosome 9, is a frequent event in glioma and has been associated with poor prognosis [19,20,21].

In one PCL (PCL10), a PCR product was obtained for the *CDKN2A^INK4a^* promoter but not for *CDKN2A* exon 2. The promoter was unmethylated in this sample. Notably, a product was also generated for *CDKN2B* exon 2. HRM and PSQ data revealed that *CDKN2B* exon 2 was highly methylated. This finding demonstrates that our novel method successfully amplified the target region in originally methylated DNA despite the presence of three mispriming sites in the reverse primer.

For one PCL (PCL16), we obtained a PCR product for *CDKN2A* exon 2 but not for the promoter, suggesting a partial gene deletion. Interestingly, *CDKN2A* exon 2 was largely unmethylated, except for CpG 21, which was highly methylated (87.9%). Such isolated hypermethylation in an otherwise unmethylated region may result from aberrant de novo methylation and could indicate a regulatory role of CpG 21. In general, methylation of a single CpG can affect transcription factor binding, nucleosome positioning, or RNA splicing. However, the biological relevance of CpG 21 hypermethylation in this context remains unclear.

In nine PCLs (PCL10, PCL12, PCL13, PCL17-19, PCL23, PCL25, PCL27), the *CDKN2A^INK4a^* promoter was unmethylated (cut-off 8%), while in eight PCLs (PCL11, PCL14, PCL15, PCL20-22, PCL24, PCL26), it showed slight methylation. In two of these samples (PCL11, PCL12), the ratio of PCR products for *CDKN2A* exon 2 and *CDKN2B* exon 2 was <80%. Thus, reliable methylation levels were only obtained for five CpGs—those that are specific for *CDKN2A* exon 2 and *CDKN2B* exon 2, as described above for mixtures of methylated and unmethylated DNA standards. In three samples (PCL13–PCL15), the proportion of *CDKN2A* exon 2 was between 80% and 90%. These samples showed moderate and relatively heterogeneous methylation across the 24 CpGs. In twelve PCLs (PCL16–PC27), the proportion of *CDKN2A* exon 2 was >90%, and these samples generally showed high methylation of *CDKN2A* exon 2.

In all PCLs analyzed in this study, mean *CDKN2A^INK4a^* promoter methylation was <12%. While high promoter methylation is commonly associated with transcriptional silencing of *CDKN2A* in glioma [22], low or moderate methylation levels may result in partial inactivation. However, due to the lack of expression data for this sample set, the functional consequences of low/moderate promoter methylation and exon 2 methylation remain speculative.

To address this limitation, we next examined the relationship between *CDKN2A* exon 2 methylation and gene expression in commercial breast cancer cell lines for which DNA, RNA, and protein fractions were available.

### 2.5. DNA Methylation Analysis of the CDKN2A^INK4a^ Promoter and CDKN2A Exon 2 and Transcript Variant Analysis of CDKN2A Exons in Commercial Breast Cancer Cell Lines

**Figure 9:**
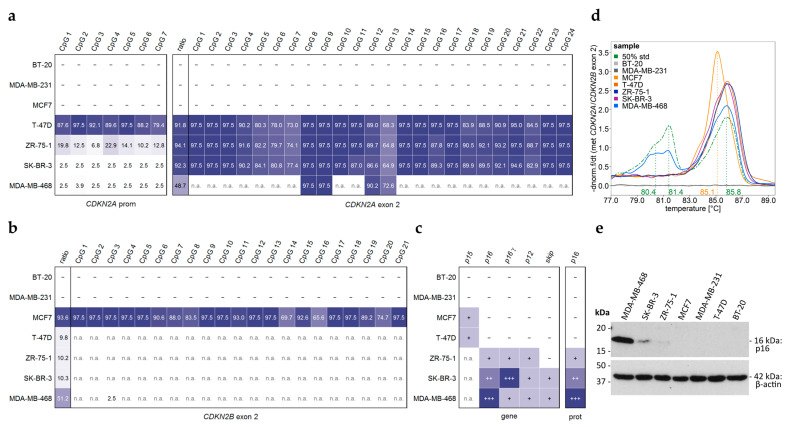
Heatmap for the *CDKN2A^INK4a^* promoter and *CDKN2A*/*CDKN2B* exon 2 methylation levels of commercial cell lines (**a**) *CDKN2A*, (**b**) *CDKN2B*. (**c**) Gene expression of *CDKN2A* and *CDKN2B* transcripts as well as p16^INK4a^ protein expression determined by (**e**) Western blotting. (**d**) Negative derivative of normalized HRM curves for *CDKN2A*/*CDKN2B* exon 2 methylation. —not present/detected, intensity of expression from + to +++. n.a.: not analyzable due to simultaneous determination of methylation levels of *CDKN2A* and *CDKN2B*.

**Figure 10:**
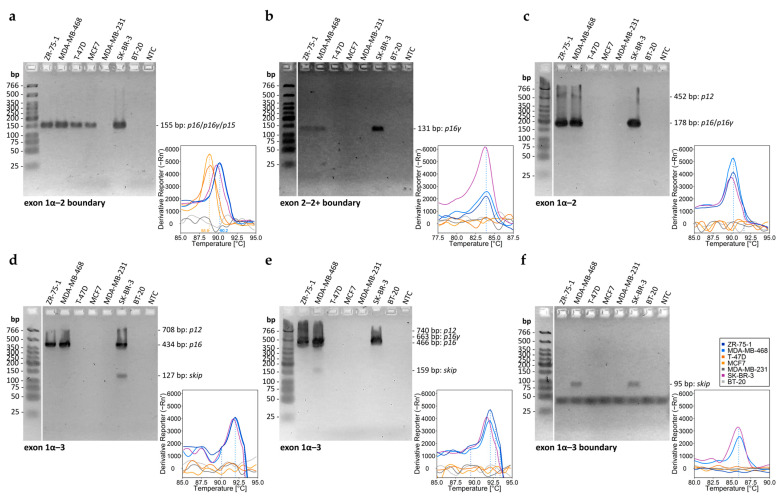
Identification of *CDKN2A^INK4a^*/*CDKN2B^INK4b^* transcripts by (**a**–**f**) agarose gel electrophoresis and melt curve analysis in commercial cell lines by applying different primer sets. Dotted lines: melting temperatures. The expected product length is shown on the right of the gel.

Seven commercial breast cancer cell lines (BT-20, MCF7, MDA-MB-231, T-47D, ZR-75-1, SK-BR-3, and MDA-MB-468) were analyzed for *CDKN2A^INK4a^* promoter and *CDKN2A* exon 2 methylation (Figure 9a–d). Unlike sample set 1, RNA and protein fractions were also available for these cell lines, enabling us to investigate potential associations between the DNA methylation status of the *CDKN2A^INK4a^* promoter and *CDKN2A* exon 2 and gene transcription at both the mRNA and protein levels (Figure 9 and Figure 10).

In breast cancer, the role of p16^INK4a^ is less clearly defined than in other tumor types, as both deletions at 9p21 and overexpression of p16^INK4a^ have been proposed as markers of breast cancer progression [23]. The relevance of *CDKN2A^INK4a^* promoter methylation in breast cancer remains controversial [9,10,11,12,13,14], whereas hypermethylation of *CDKN2A* exon 2 methylation has been linked to breast tumorigenesis [16].

For two cell lines, BT-20 and MDA-MB-231, no PCR products were obtained for either *CDKN2A^INK4a^* promoter or *CDKN2A*/*CDKN2B* exon 2 in the DNA methylation assays (Figure 9a). Correspondingly, no *CDKN2A^INK4a^* transcripts were detected (Figure 9c and Figure 10). Our results support the literature [24] and ATCC [25] data that report homozygous deletion of *CDKN2A* in these cell lines. In addition, we did not obtain *p15* (*CDKN2B^Ink4b^*) transcripts in these two cell lines.

MCF-7 also showed no PCR products for the *CDKN2A^INK4a^* promoter, *CDKN2A* exon 2, nor any *CDKN2A* transcripts (Figure 9a,c and Figure 10), consistent with a homozygous deletion. However, a PCR product was obtained for *CDKN2B* exon 2, which was highly methylated (Figure 9b). Notably, the melting temperature of the *CDKN2B* product was 0.7 °C lower than that of the PCR product for methylated *CDKN2A* exon 2 (Figure 9d), reflecting a slightly lower GC content (47.8% vs. 48.7%) after bisulfite conversion. We also detected a transcript of *p15* (*CDKN2B^Ink4b^*), inferred by exclusion: when transcripts for neither *p16* nor *p16γ* were detected, and the melting temperature corresponded to the GC content of *p15* (88.8° C, 70.3% GC) rather than *p16*/*p16γ* (90.2 °C, 71.6% GC), the product from the exon 1α–exon 2 boundary primer set was attributed to *p15* (Figure 10).

In T-47D, ZR-75-1, and SK-BR-3, PCR products for both *CDKN2A^INK4a^* promoter and *CDKN2A* exon 2 were detected. The proportion of *CDKN2A* exon 2 product exceeded 90%, enabling reliable DNA methylation analysis. All three cell lines exhibited high *CDKN2A* exon 2 methylation but differed in promoter methylation: high in T-47D, intermediate in ZR-75-1, and absent in SK-BR-3. Transcripts of *CDKN2A* and p16^INK4a^ expression were detected in ZR-75-1 and SK-BR-3 but not in T-74D (Figure 9e). In T-74-D, *p15* (*CDKN2B^Ink4b^*) was detected instead. Results shown in Figure 10d–f suggest exon 2 skipping in SK-BR-3.

In MDA-MB-468, the *CDKN2A^INK4a^* promoter was unmethylated. PCR amplification resulted in a high proportion (51.2%) of *CDKN2B* product, limiting reliable methylation analysis to four CpGs, which were highly methylated. Our results suggest exon 2 skipping in MDA-MB-468. Transcripts of *CDKN2A* and p16^INK4a^ expression were detected (Figure 9e). Differentiation of p16^INK4a^ expression from the formation of other *CDKN2A* transcripts required multiple SYBR green-based PCRs, agarose gel electrophoresis, and melt curve analysis.

*CDKN2A* (and *CDKN2B*) inactivation, via deletion or epigenetic silencing, has been associated with breast cancer [9,10,11,12,13,26,27]. In our study, BT-20 and MDA-MB-231 showed complete inactivation of both genes via homozygous deletion. In MCF7 and T-47D, *CDKN2A* transcripts were absent, but *p15* (*CDKN2B^Ink4b^*) was detected, suggesting a compensatory role for *CDKN2B^Ink4b^*. This is in line with a mouse study hypothesizing that p15^INK4b^ can substitute for p16^INK4a^ in stress conditions, possibly explaining their frequent co-deletion in cancer [28].

In contrast, ZR-75-1, SK-BR-3, and MDA-MB-468 expressed multiple *CDKN2A* transcripts by having high *CDKN2A* exon 2 methylation. However, promoter methylation was different: intermediate in ZR-75-1, absent in SKBR3 and MDA-MB-468. These observations suggest that high promoter methylation is associated with transcriptional silencing, whereas intermediate or low methylation does not preclude gene expression. Notably, neither promoter nor exon 2 methylation consistently predicted mRNA or protein levels, indicating that regulation of *CDKN2A* gene expression is even more complex.

Our cell line panel comprised three breast cancer molecular subtypes: luminal A (MCF7, T-47D, and ZR-75-1), HER-2-positive (SK-BR-3), and triple negative (BT-20, MDA-MB-231, and MDA-MB-468). BT-20 and MDA-MB-231, both triple negative, lacked *CDKN2A* due to homozygous deletion, while MDA-MB-468 (also triple negative) highly expressed *CDKN2A*. Among the luminal A cell lines, MCF7 and T-47D expressed *p15* (*CDKN2B^Ink4b^*), whereas ZR-75-1 expressed p16*^Ink4a^*. These results suggest that *CDKN2A* is not regulated in a molecular subtype-specific manner. This variability suggests that *CDKN2A* regulation is not strictly molecular subtype-specific.

Previous studies have linked breast cancer subtypes to distinct DNA methylation patterns [29,30]. We observed high *CDKN2A* exon 2 methylation in luminal A (T-47D, ZR-75-1), HER2-positive (SK-BR-3), and triple-negative (MDA-MB-468) cell lines. Promoter methylation varied: high in T-47D, intermediate in ZR-75-1, and absent in SK-BR-3 and MDA-MB-468. These findings suggest that neither *CDKN2A^INK4a^* promoter nor *CDKN2A* exon 2 methylation follows a clear subtype specific pattern. However, a recent study reported *CDKN2A^INK4a^* promoter hypermethylation in triple-negative compared to non-triple-negative breast cancer patients [31].

Our approach provides a practical tool for the locus-specific analysis of *CDKN2A* exon 2 in cancer despite the high sequence identity with *CDKN2B*. Since *CDKN2A* methylation is a well-established biomarker in several tumor types, including glioblastoma and melanoma, reliable discrimination between *CDKN2A* and *CDKN2B* is critical for both research and diagnostic applications. The combined analysis of methylation and transcript variants from the same exon region may also provide insights into epigenetic regulation and transcript diversity with potential clinical relevance.

One limitation of our approach is the need to estimate *CDKN2A*-specific methylation levels based on nucleotide differences at at least one position within the target sequence. Although PSQ is widely considered a reliable and quantitative method for DNA methylation analysis, sequence context and assay design can influence signal intensities, and minor variability cannot be excluded. We did not identify a restriction enzyme that selectively digests *CDKN2B* exon 2 while leaving *CDKN2A* intact, which would be a theoretically attractive strategy to improve locus specificity.

Long-read DNA nanopore of RNA sequencing (RNA-seq) as well as whole-genome bisulfite sequencing (WGBS) and short-read RNA-seq using 2 × 150 bp paired-end reads would allow for a comprehensive, genome-wide assessment of DNA methylation and alternative splicing events. However, these approaches require substantial sequencing depth and bioinformatic resources, particularly when aiming to analyze regions with high sequence identity such as *CDKN2A* and *CDKN2B* exon 2. In our study, we deliberately chose targeted approaches for DNA methylation and transcript variant analysis to accurately discriminate between these highly homologous regions. Despite the challenges associated with primer design, these methods enable precise locus-specific analysis and are more readily applicable when large-scale sequencing is not feasible.

## 3. Materials and Methods

### 3.1. Primary Cell Lines from Glioma Patients

The sample set consisted of primary human tumor cell lines (PCLs) established from 27 glioma patients as described previously [32]. The study was approved by the local Ethics Commission of the Faculty of Medicine at the Johannes Kepler University Linz (application number E-39-15). All patients signed a written informed consent form.

### 3.2. Commercial Breast Cancer Cell Lines

Seven breast cancer cell lines were obtained from ATCC (American Type Culture Collection; Manassas, VA, USA). The molecular subtypes were luminal A (MCF7, T-47D, and ZR-75-1), Her2-positive (SK-BR-3), and triple negative (BT-20, MDA-MB-231, and MDA-MB-468). Cells were cultured as described previously [18]. Cells were harvested at ~70% confluency and cell pellets were stored at −80 °C until DNA extraction.

### 3.3. DNA/RNA Extraction and Bisulfite Conversion

Genomic DNA and RNA (only from commercial breast cancer cell lines) were isolated simultaneously using an AllPrep DNA/RNA Mini Kit (Qiagen, Hilden, Germany) following the manufacturer’s protocol for cultured cells and quantified with a Nanodrop 2000c spectrophotometer (Thermo Fisher Scientific, Vienna, Austria). The DNA and RNA extracts were stored at −20 °C until reverse transcription (RNA) or bisulfite conversion (DNA).

Reverse transcription of RNA was performed with a QuantiTect Reverse Transcription Kit (Qiagen) following the manufacturer’s instructions. cDNA was stored at −20 °C until real-time PCR.

For bisulfite conversion, the EpiTect Fast Bisulfite Conversion Kit (Qiagen, Hilden, Germany) was used according to the manufacturer’s protocol. The converted DNA was quantified using the Qubit 4 instrument with a Qubit ssDNA Assay Kit (Thermo Fisher Scientific, Vienna, Austria) and stored at −20 °C until PCR.

### 3.4. Primer Design and PCR Conditions for DNA Methylation Analysis

The assays were developed in-house. Nucleotide sequences were retrieved from the National Center for Biotechnology Information (NCBI) database [17]. NCBI reference sequences were used for exon annotation (*CDKN2A p16^Ink4a^* transcript: NM_000077.5; *CDKN2B p14^Ink4b^* transcript: NM_004936.4). For the *CDKN2A p16^Ink4a^* promoter, the seven CpGs upstream of exon 1α were targeted. For exon 2, CpGs 1–24 and CpGs 1–21 of the *CDKN2A* and *CDKN2B* gene were targeted, respectively. The CpGs of exon 2 are part of CpG islands according to the University of California at Santa Cruz (UCSC) Genome Browser [33] (*CDKN2A*: Genbank GRCh38.p13 chr9 NC_000009.12:c21970915–21971191, 35 CpGs; *CDKN2B*: Genbank GRCh38.p13 chr9 NC_000009.12:c22005889–22006230, 35 CpGs). Primers were designed with PyroMark Assay Design Software 2.0.1.15 (Qiagen, Hilden, Germany) and purchased from Sigma-Aldrich (Steinheim, Germany). The primer sequences are given in Table 1 and their location is visualized in Figure 1.

**Table 1:** Overview of HRM-PSQ assays.

Region	Primer Sequence (5′→3′)	Length [bp]	CpGs Analyzed	
			***CDKN2A***	***CDKN2B***
promoter	F: GAGGGGTTGGTTGGTTATTAG	75		
	R: [Btn] TACCTACTCTCCCCCTCTC			
	S: GGTTGGTTGGTTATTAGA		1–7	-
exon 2	F: GTTTTTTTTGGTAGGTTATGATGATGG	230		
	R: [Btn] ACCCAACTCCTCAACCAAATCC			
	S1: AGGTTATGATGATGGGTA		1–14	1–11
	S2: GGGAGGGTTTTTTGGATA		15–24	12–21

[Btn]: biotin, length: PCR product length; bp: base pairs, F: forward primer, R: reverse primer, and S: sequencing primer.

For each primer set, concentration and annealing temperature (Ta) were optimized by using bisulfite-converted in-house-prepared unmethylated and methylated control DNA. In-house preparation by whole-genome amplification and enzymatic in vitro methylation was performed as described previously [34], where all details of the protocol are provided.

Each reaction was performed in a total volume of 20 μL, consisting of 1× PCR mix, forward and reverse primer, and 5 ng of bisulfite converted DNA using a Rotor-Gene Q instrument with a 72-well rotor (Qiagen, Hilden, Germany). For the exon 2 region, 400 nM of each primer with 10 µL PyroMark PCR Master Mix (2×), 2 µL CoralLoad Concentrate (10×), and 1 µL EvaGreen dye (20×) (Biotium, Fremont, CA, USA) were used for HRM-PSQ. For the promoter region, 400 nM of each primer with either 10 µL PyroMark PCR Master Mix (2×) and 2 µL CoralLoad Concentrate (10×) were used for PSQ or 10 µL EpiTect HRM Master Mix (2×) for HRM. Amplification was performed with initial activation at 95 °C for 15 min (PyroMark PCR Master Mix) or 5 min (EpiTect HRM Master Mix), followed by 50 cycles of denaturation at 94 °C for 30 s, annealing at 62.1 °C (exon 2) or 59.5 °C (promoter) for 30 s, elongation at 72 °C for 30 s, and final elongation at 72 °C for 10 min.

For HRM, the following program was applied directly after final elongation: strand separation for 1 min at 95 °C, strand hybridization for 1 min at 40 °C and HRM with a ramp from 65 °C to 95 °C with 0.1 °C/hold (2 s) and gain optimization (70% before melt).

Each PCR run included bisulfite-converted human non-methylated and methylated DNA; 25%, 50%, and 75% mixtures thereof; and a no template control (2 μL nuclease-free H_2_O). Identity, quality, and yield of PCR products obtained for standards were assessed by gel electrophoresis (3% agarose gel in 1× TBE (Tris-borate-EDTA) buffer. The gel was post-stained with 3× GelRed (Biotium, Fremont, CA, USA), and bands were visualized with a UVT-20 M transilluminator (Herolab, Wiesloch, Germany).

### 3.5. PSQ of PCR Products

For PSQ, the PyroMark Q24 Vacuum Workstation, the PyroMark Q24 Advanced instrument with PyroMark Q24 Advanced Accessories, PyroMark Q24 Advanced CpG Reagents (all Qiagen, Hilden, Germany), and Sepharose High-Performance beads (GE Healthcare, Vienna, Austria; Thermo Fisher Scientific, Vienna, Austria) were used according to the manufacturer’s instructions.

For the promoter and exon 2 (sequencing primer 1), the dispensation order was adapted, e.g., to overcome sequencing frameshifts. The dispensation orders were as follows: promoter: AGCTGGTCGTATCTAGTCGTAGTCAGTCTAGTCGTAGTCGAG; exon 2 S1: AGCTAGTCAGACTGTAGCTGAGCTGTGTGTTGTACTAGCTAGCTGAGTATATGCTAGTACGTATTCGTATTGTATCGTATCGTGATGACTGTACTGTAG-TCGAGTTGATGACTGTGTGTGTGTGATCAGTCGGCTAGCTGTGTACTGTAGCTAG-CTGATGTGGTCGTAGTCGTG; exon 2 S2: ACTGTGTGTGTGCTGTATCAGGTCGGCT-AGCTGTGTACTGTAGCTAGCTGATGTGGTCGTAGTCGTG).

For the promoter region, 1 μL Streptavidin Sepharose High Performance (GE Healthcare, Vienna, Austria), 40.0 μL PyroMark Binding Buffer, 24.0 µL high-purity water (18.2 MΩ cm, ELGA PURELAB Ultra MK 2, Veolia, Celle, Germany), and 15.0 μL of biotinylated PCR product were mixed by agitating for 10 min at 1400 rpm. For each sequencing primer for the exon 2 region, 0.6 μL Streptavidin Sepharose High Performance (GE Healthcare, Vienna, Austria), 24.0 μL PyroMark Binding Buffer, and 14.4 µL high-purity water (18.2 MΩ) were mixed with 9.0 μL of biotinylated PCR product (same PCR well for both sequencing primers). All following steps were performed as described previously [35].

### 3.6. Analysis of Transcript Variants

All real-time-PCR assays for analysis of transcript expression were developed in-house. NCBI reference sequences [17] were used for exon annotation (*CDKN2A*: *p16^Ink4a^* transcript NM_000077.5, *p16γ^Ink4a^* transcript NM_001195132.2, *p12^Ink4a^* transcript NM_058197.5, *p14^ARF^* transcript, *tr6^ARF2^* transcript NM_001363763.2, *CDKN2B*: *p14^Ink4b^* transcript NM_004936.4, and *p10^Ink4b^* transcript NM_078487.2). Primers were designed with the web interface Primer3Plus [36] and purchased from Sigma-Aldrich (Steinheim, Germany).

Primer sets 1–3 (Table 2) target *CDKN2A* exon–exon boundaries, whereas primers from sets 4–6 (Table 3) do not bind exon–exon boundaries. Transcript variants targeted by the respective primer set are shown in Figure 2.

**Table 2:** Primer sets for gene expression analysis targeting *CDKN2A* exon–exon boundaries.

Set	Region	Primer Sequence (5′→3′)	Location	Length [bp]
1	exon 1α–exon 2	F: GGAGGCCGATCCAGGTCA	boundary	155
		R: CAGCACCACCAGCGTGTC	exon 2	
2	exon 2–exon 2+	F: GCGGAAGGTCCCTCAGAA	boundary	131
		R: CAGCCAGCTTGCGATAACCA	exon 2+	
3	exon 1α–exon 3	F: GATCCAGACATCCCCGATTG	boundary	95
		R: CCTGTAGGACCTTCGGTGA	exon 3	

bp: base pairs, F: forward primer, R: reverse primer.

**Table 3:** Primer sets for gene expression analysis targeting *CDKN2A* exons.

Set	Region	Primer Sequence (5′→3′)	Location	Length [bp]
4	exon 1α–exon 2	F: CAACGCACCGAATAGTTACG	exon 1α	178/452
		R: CAGCACCACCAGCGTGTC	exon 2	
5	exon 1α–exon 3	F: CAACGCACCGAATAGTTACG	exon 1α	434/631/
		R: CAGTTGTGGCCCTGTAGGA	exon 3	708/127
6	exon 1α–exon 3	F: GGTCGGGTAGAGGAGGTG	exon 1α	466/663/
		R: AGGACCTTCGGTGACTGATGA	exon 3	740/159

bp: base pairs, F: forward primer, R: reverse primer.

Each reaction was performed in a total volume of 20 µL, consisting of 10 µL QuantiTect SYBR Green PCR Master Mix, RNase-free water, forward and reverse primer (final concentration of 300 nM each), and 10 ng cDNA using the QuantStudio 5 instrument (Thermo Fisher Scientific, Vienna, Austria) and fast ramp speed. The temperature program included an initial activation step of 15 min at 95 °C, followed by a repeated 3-step cycling of 10 s denaturation at 94 °C, annealing for 20 s at 60 °C, and elongation at 72 °C. For primer sets targeting exon–exon boundaries, 40 cycles and an elongation time of 20 s were used. For primer sets targeting exons, 45 cycles and 45 s elongation were applied. Subsequent melt curve acquisition was performed from 60 °C to 95 °C in 0.1 °C/s steps. For each primer set assay, the identity and purity of the PCR products were checked by gel electrophoresis (3% agarose gel in TBE buffer and post-staining using 3× GelRed (Biotium, Fremont, CA, USA) and a UVT-20 M transilluminator (Herolab, Wiesloch, Germany)).

### 3.7. Western Blot Analysis

Analysis of p16^Ink4a^ expression, cell fractionation, protein separation, and Western blotting were performed as described previously [37]. The following antibodies were used: rabbit anti-p16ink4a (RM267, Sigma Aldrich, Steinheim, Germany) dilution 1:1000, mouse anti-beta actin (Sigma Aldrich, Steinheim, Germany) dilution 1:2000. The anti-mouse IgG (#A0168; 1:10,000) horseradish peroxidase (HRP)-coupled secondary antibody was purchased from Sigma-Aldrich (Steinheim, Germany), and the anti-rabbit IgG (#7074; 1:5000) HRP-linked secondary antibody was obtained from Cell Signaling Technology (Danvers, MA, USA).

### 3.8. Data Analysis

Melting curves obtained by PCR-HRM were assessed and exported using Rotor-Gene Q Series Software 2.3.1 (Qiagen, Hilden, Germany). PSQ data was evaluated and exported with PyroMark Q24 Advanced software 3.0.0 (Qiagen, Hilden, Germany). Exported data was analyzed and presented graphically using R version 3.6.2 [38]. The R-packages used, including corrplot, ggplot2, and polynom, are listed in the Supplementary Materials R-packages.

For HRM analysis, derivative melting curves were calculated from normalized melting curves by applying Savitzky–Golay filtering for third-degree polynomials.

DNA methylation levels of the promoter region obtained by HRM were calculated from normalized melting curves using temperature-wise calibration as described previously [34]. Polynomial grade 3 calibration functions were calculated from standards with a methylation status of 0–75% for the temperatures 79.6–81–6 °C and applied on normalized fluorescence data from samples.

DNA methylation levels and nucleotide ratios obtained by PSQ ≤ 5.00% (lower limit of quantification, LLOQ) and ≥95.00% (upper limit of quantification, ULOQ) were substituted with default values, namely 2.50% and 97.50%, respectively [39].

## 4. Conclusions

In this study, we established a novel strategy to reliably determine the methylation status of *CDKN2A* exon 2 despite its high sequence similarity with *CDKN2B*. The target region for *CDKN2A* and *CDKN2B* was co-amplified using a novel primer set. By evaluating the ratio of *CDKN2A* and *CDKN2B* products, DNA methylation levels for *CDKN2A* exon 2 could be determined accurately.

This approach was successfully applied to a panel of breast cancer and glioma cell lines, revealing diverse genetic and epigenetic alterations of *CDKN2A*. In particular, we identified homozygous deletions, transcript-specific expression patterns, and instances of exon 2 skipping. Our findings underscore the complexity of *CDKN2A* regulation in cancer and demonstrate that neither promoter nor exon 2 methylation alone is predictive of gene expression. Importantly, the lack of consistent subtype-specific methylation patterns in breast cancer cell lines highlights the need for a deeper understanding of *CDKN2A* silencing mechanisms. This method can support future studies aiming to dissect the functional consequences of *CDKN2A* alterations in various tumor types.

## Data Availability

All data generated during this study are included in this published article. Further inquiries can be directed to the corresponding author.

## Supplementary Materials

The following supporting information can be downloaded at: https://www.mdpi.com/article/10.3390/ijms26136128/s1.
