# Supplementary material for: DNA Methylation and Transcript Variant Analysis of CDKN2A Exon 2 Despite High Sequence Identity with CDKN2B Exon 2
Source: Int J Mol Sci. 2025 Jun 26;26(13):6128. doi: 10.3390/ijms26136128 (PMC12250293; doi:10.3390/ijms26136128)
Supplement: Supplementary file 1 [file ijms-26-06128-s001.zip › ijms-3665617-supplementary.pdf]

Supplementary R-packages

DNA Methylation and Transcript Variant Analysis of *CDKN2A* Exon 2 Despite High Sequence Identity with *CDKN2B* Exon 2

Katja Zappe [1], Andreas Jenik [1], Daniel Berger [1], Lukas Uhlik [2], Petra Heffeter [2] and Margit Cichna-Markl [1,*]

[1] Department of Analytical Chemistry, Faculty of Chemistry, University of Vienna, 1090 Vienna, Austria; katja.zappe@univie.ac.at (K.Z.); a.jenik@hlw19.at (A.J.); office@berger-daniel.at (D.B.)
[2] Center for Cancer Research and Comprehensive Cancer Center, Medical University of Vienna, 1090 Vienna, Austria; lukas.uhlik@gmx.at (L.U.); petra.heffeter@meduniwien.ac.at (P.H.)
* Correspondence: margit.cichna@univie.ac.at

David Robinson, Alex Hayes and Simon Couch (2022). broom: Convert Statistical Objects into Tidy Tibbles. R package version 1.0.1. https://CRAN.R-project.org/package=broom

John Fox and Sanford Weisberg (2019). car: An {R} Companion to Applied Regression, Third Edition. Thousand Oaks CA: Sage. URL: https://socialsciences.mcmaster.ca/jfox/Books/Companion/

Winston Chang (2014). extrafont: Tools for Using Fonts. R package version 0.17. https://CRAN.R-project.org/package=extrafont

Hadley Wickham (2021). forcats: Tools for Working with Categorical Variables (Factors). R package version 0.5.1. https://CRAN.R-project.org/package=forcats

Luke Smith (2016). ggloop: Create 'ggplot2' Plots in a Loop. R package version 0.1.0. https://github.com/seasmith/ggloop

Hadley Wickham (2016). ggplot2: Elegant Graphics for Data Analysis. Springer-Verlag New York. https://ggplot2.tidyverse.org

Alboukadel Kassambara (2020). ggpubr: 'ggplot2' Based Publication Ready Plots. R package version 0.4.0. https://CRAN.R-project.org/package=ggpubr

Gregory R. Warnes, Ben Bolker and Thomas Lumley (2021). gtools: Various R Programming Tools. R package version 3.9.2. https://CRAN.R-project.org/package=gtools

Stefan Milton Bache and Hadley Wickham (2020). magrittr: A Forward-Pipe Operator for R. R package version 2.0.1. https://CRAN.R-project.org/package=magrittr

Henrik Bengtsson (2022). matrixStats: Functions that Apply to Rows and Columns of Matrices (and to Vectors). R package version 0.62.0. https://CRAN.R-project.org/package=matrixStats

Simon Wood (2017) Generalized Additive Models: an introduction with R (2nd edition), CRC, New York. https://CRAN.R-project.org/package=mgcv

Philipp Schauberger and Alexander Walker (2021). openxlsx: Read, Write and Edit xlsx Files. R package version 4.2.4. https://CRAN.R-project.org/package=openxlsx

Kevin Wright (2021). pals: Color Palettes, Colormaps, and Tools to Evaluate Them. R package version 1.7. https://CRAN.R-project.org/package=pals

Bill Venables and Kurt Hornik and Martin Maechler (2019). polynom: A Collection of Functions to Implement a Class for Univariate Polynomial Manipulations. R package version 1.4-0. https://CRAN.R-project.org/package=polynom

Alboukadel Kassambara (2021). rstatix: Pipe-Friendly Framework for Basic Statistical Tests. R package version 0.7.0. https://CRAN.R-project.org/package=rstatix

Hadley Wickham and Dana Seidel (2020). scales: Scale Functions for Visualization. R package version 1.1.1. https://CRAN.R-project.org/package=scales

signal developers (2013). signal: Signal processing. http://r-forge.r-project.org/projects/signal/

Hadley Wickham (2019). stringr: Simple, Consistent Wrappers for Common String Operations. R package version 1.4.0. https://CRAN.R-project.org/package=stringr

Wickham et al., (2019). Welcome to the tidyverse. Journal of Open Source Software, 4(43), 1686. https://doi.org/10.21105/joss.01686

Simon Garnier, Noam Ross, Robert Rudis, Antônio P. Camargo, Marco Sciaini, and Cédric Scherer (2021). Rvision - Colorblind-Friendly Color Maps for R. R package version 0.6.2. https://sjmgarnier.github.io/viridis/
